# Relationship between Vitamin D Receptor Gene Polymorphisms and Migraine without Aura in an Iranian Population

**DOI:** 10.1155/2013/351942

**Published:** 2013-07-25

**Authors:** Mohammad Motaghi, Shaghayegh Haghjooy Javanmard, Faraidoon Haghdoost, Mohamadhasan Tajadini, Mohammad Saadatnia, Laleh Rafiee, Alireza Zandifar

**Affiliations:** ^1^Physiology Research Center, Department of Physiology, Isfahan University of Medical Sciences, Isfahan, Iran; ^2^Medical Student Research Center, Isfahan University of Medical Sciences, Isfahan 81745-319, Iran; ^3^School of Pharmacy and Isfahan Pharmaceutical Sciences Research Center, Isfahan University of Medical Sciences, Isfahan, Iran; ^4^Department of Neurology and Isfahan Neurosciences Research Center, Isfahan University of Medical Sciences, Isfahan, Iran

## Abstract

*Background*. Inflammation has a key role in migraine pathophysiology. Vitamin D is an effective anti-inflammatory agent. The aim of this study was to investigate the association between migraine and two vitamin D receptor (VDR) polymorphisms (TaqI and FokI) and also the relationship between VDR polymorphisms and headache severity. *Methods*. In this case-control study we assessed 103 patients with newly diagnosed migraine without aura and 100 healthy subjects. Patients filled headache impact test-6 (HIT-6) as a tool to assess headache severity. *Results*. Genotype frequencies of VDR were significantly different between control and migraine patients. Heterozygote genotypes (Ff and Tt) were statistically more frequent in the migraine patients than the control subjects both for TaqI gene (*P* = 0.018; OR = 1.81, 95% CI = 1.03–3.18) and FokI gene polymorphisms (*P* = 0.001; OR = 2.91, 95% CI = 1.47–5.77). Also f and t alleles were more frequent in the migraine patients. Total HIT-6 score was significantly different between FokI heterozygote and homozygote patients (60.32 ± 1.87 versus 49.87 ± 2.69, resp., *P* = 0.004). *Conclusions*. In conclusion our results showed that TaqI and FokI gene polymorphisms are associated with migraine without aura in Iranians patients. Also headache severity in FokI heterozygote patients was significantly greater than in the homozygote patients.

## 1. Introduction

Migraine is a major cause of chronic headaches with a reported prevalence of about 17% in women and 6% in men. Migraine is clinically diagnosed by recurrent unilateral pulsatile headaches and may happen after motor or sensory symptoms. The headache in migraine patients could be accompanied by nausea, vomiting, phonophobia, and photophobia. So, these migraineurs will have a low quality of life and less efficacy at work. Migraine has been ranked as the 8th most debilitating disease [1, 2].

To find a comprehensive explanation for the underlying pathophysiology of migraine as a disease, attempts have come to form some theories which can somehow justify the heterogeneity of the symptoms. One of these hypotheses is the role of inflammation in physiopathology of migraine. It has been shown that inflammatory substances produced by mast cells, especially in meninges, can activate the trigeminal nerve which is a central event in the pain of migraine [3]. Also, people with allergies may have more migraine attacks in some seasons, which may point to a role of inflammation [4].

On the other hand vitamin D has important functions in lots of physiologic activities such as immune system regulation and resolution of inflammation [5]. Furthermore, vitamin D has a key role in regulating cell mediated immunity (CMI) [5]. CMI is a source for inflammatory mediators. It has been demonstrated that vitamin D deficiency could result in a proinflammatory state and more interleukins release [6]. Although the effect of vitamin D in some pathological conditions, for instance, chronic pains, is yet to be proved, there is a need for more supporting studies [7].

Vitamin D is suggested to play a role in serotonin [8] and dopamine release [9] which are both proposed as neurotransmitters involved in the pathogenesis of migraine. The presence of vitamin D receptors (VDRs) in the hypothalamus which is a region for migraine pain sensation [8] can support the hypothesis of vitamin D contribution in migraine.

VDR is a nuclear protein; VDR gene is located on chromosome 12q and has several polymorphisms. Four known single nucleotide polymorphisms at the 3′ end of this gene are FokI, TaqI, ApaI, and BmsI. The FokI polymorphism, also known as start codon polymorphism, is caused by replacement of C with T and makes the final protein shorter.

The aim of this study was to find whether there is an association between two VDR polymorphisms (TaqI and FokI) and migraine without aura.

## 2. Materials and Methods

### 2.1. Patients and Settings

This case-control study included 103 consecutive patients who were newly diagnosed as having migraine without aura, based on International Headache Society (IHS) criteria [10] from the outpatient neurology clinics of three hospitals in Isfahan, Iran, between April 2011 to June 2011. The sociodemographic and headache characteristics of all the subjects including age, sex, frequency of headaches, family history, and menstrual effect on the headache aggravation were asked. Controls were selected from healthy people who accompanied the patients that were referred to the neurologic clinics (including patients with migraine and other neurologic disorders) who did not have any history of migraine and also did not have any family history of migraine in first degree relatives. We did not select any accompanying people who had genetic relation with migraine patients. Informed consent was obtained from the participants before inclusion in the study. The study was approved by the Ethics Committee of Isfahan University of Medical Sciences.

### 2.2. HIT-6 Questionnaire

Headache impact test-6 (HIT-6) questionnaire was filled out by all patients to assess headache severity. The HIT-6 questionnaire consists of six questions that show six dimensions: pain severity, role functioning, social functioning, vitality, emotional distress, and cognitive functioning. Each question has 5 available answers: never, rarely, sometimes, very often, and always. Choices have been scored between 6 and 13, respectively. The total score is between 36 and 78 [11].

### 2.3. DNA Extraction and Genotyping

Two mL of peripheral blood was collected from each participant. Genomic DNA samples were extracted from peripheral whole blood, using the AccuPrep Genomic DNA Extraction kit (Bioneer Inc., Republic of Korea) according to the manufacturer's protocol.

The single nucleotide polymorphisms (SNPs) rs2228570 and rs731236 were identified by the *NCBI* data bank, and primers were designed by Beacon Designer 7.91 to flank the coding regions (PREMIER Biosoft International, USA, and synthesized by TIB MOLBIOL, Germany). The sequences of primers are shown in Table 1.

Genotyping was done by high-resolution melt (HRM) assay using a Rotor-Gene 6000 instrument (Corbett Life Science, Australia).

PCR reactions were carried out in duplicate in 20 *μ*L of final volume using the type-it HRM kit (Qiagen), HRM PCR buffer, HotStarTaq *Plus* DNA Polymerase, nucleotides and EvaGreen dye, and 30 ng DNA.

The PCR program consisted of an initial denaturation-activation step at 95°C for 5 min, followed by a 40-cycle program (denaturation at 95°C for 15 s, annealing conditions 55°C for 5 seconds, 72°C for 15 seconds; an HRM step from 70 to 95°C rising at 0.1°C per second).

Curves for each duplicate were checked on the shape and peak height to meet reproducibility.

Normalized and temperature-shifted melting curves from HRM, suggestive of SNP, were distinguished, and the samples were subjected to direct sequencing.

### 2.4. Data Analysis

Differences in demographics, variables, and genotypes of the VDR polymorphism variants were evaluated using a chi-squared test. An independent *t*-test was used for comparison of the quantitative variables between two groups.

The associations between VDR polymorphism and risk of migraine were estimated by computing odds ratios (ORs) and 95% confidence intervals (CIs) using logistic regression analyses and by using crude ORs. To prevent the overestimation by using OR, also relative risk (RR) was used to estimate the association between VDR polymorphism and risk of migraine.

The Hardy-Weinberg equilibrium (HWE) was tested by a goodness-of-fit chi-squared test to compare the observed genotype frequencies with the expected frequencies among controls. All statistical analyses were done with SPSS software (version 16, Chicago, IL, USA). A two-tailed *P* value of less than 0.05 was considered statistically significant.

## 3. Results

From 103 subjects with migraine without aura and 100 healthy subjects, DNA samples were analyzed for FokI rs2228570 and TaqI rs731236 polymorphisms. In the case and control group 82.6% and 78% of participants were female, respectively; and the difference was not statistically significant. Also there was no significant difference between the mean ages of case and control groups (34.08 ± 1.01 versus 34.77 ± 1.06, resp.). Patients' characteristics are reported in Table 2.

The allele frequencies of F and f for the FokI gene in the study population were 356 (87.7%) and 50 (12.3%), respectively. Frequencies in the control group were in accordance with Hardy-Weinberg equilibrium. The f allele was more frequent in the migraine patients than the control subjects (16.9% versus 7.5%, *P* = 0.002) (Table 3). For the TaqI gene, frequency of T and t alleles were 318 (78.3%) and 88 (21.7%), respectively. The t allele was more frequent in the case group than the control (25.3% versus 18%, *P* = 0.035) (Table 3). The frequencies of homozygote and heterozygote genotypes in the study population were 153 (75.4%) and 50 (24.6%) for FokI gene and 115 (56.7%) and 88 (43.3%) for TaqI gene, respectively. The heterozygote genotype was statistically more frequent in the migraine patients than the control subjects for both TaqI (50.4% versus 36%, *P* = 0.018) and FokI gene polymorphisms (33.9% versus 15%, *P* = 0.001) (Table 3). Multiple logistic regression analysis was conducted to investigate the independent role of  the FokI gene polymorphism in susceptibility to migraine and adjust the potential effect of TaqI gene polymorphism. Logistic regression analysis indicated that the Ff genotype of the FokI gene in subjects with the Tt genotype of the TaqI gene is associated with a higher risk for the disease compared with the control (OR: 3.15, 95% CI: 1.57–6.33, and *P* = 0.001).

VDR haplotypes derived from FokI and TaqI polymorphisms were examined using combination of genotypes. Subjects heterozygous for more than one polymorphic site were not considered for haplotype frequency. The distribution of haplotype frequencies in patients and control groups is shown in Table 4. Results show that frequency of TF haplotype in the control group is statistically significant more than the patients, and the haplotypes carrying t or f are more frequent in the patients group (*P* = 0.012).

The comparison of allele frequencies between males and females in the case and also control group showed that there is no significant difference in the alleles distribution according to the sex (*P* = 0.231 and 0.163 for FokI gene and *P* = 0.469 and 0.494 for TaqI gene, resp.). Further analysis in the migraine subjects showed that the distributions of FokI rs2228570 and TaqI rs731236 gene polymorphisms were not associated with the presence of a family history of migraine and also with presence of menstrual effect on the headache aggravation (*P* = 0.228 and 0.493 for FokI gene and *P* = 0.086 and 0.377 for TaqI gene, resp.). Frequency of headache per month and total HIT-6 score between the homozygote and heterozygote patients are shown in Table 5.

## 4. Discussion

In this study we found a relation between migraine and the frequency of the f allele of the FokI polymorphism, but there was no such meaningful relation with the t allele of TaqI. However we showed that TaqI heterozygote (Tt) subjects are more likely to have migraine than homozygotes (TT). In addition a significant association between FokI polymorphism heterozygotes (Ff) and migraine was shown. Furthermore we found a significantly higher HIT-6 score in the Ff genotype than in FF. Haplotype analysis showed that Tf increases the susceptibility to migraine. Logistic regression analysis indicated that Ff genotype in combination with Tt genotype is associated with a higher risk for the disease compared with the control than any other kind of genotype combination.

There are a few studies about the relationship between vitamin D and headache with controversial results. A report by Wheeler indicated that 40 percent of people with migraine had low vitamin D levels; also people with deficiencies tend to have migraine at lower ages [12]. A study by Thys-Jacobs showed alleviation of migraine with therapeutic vitamin D administration, but it investigated a small study population [13, 14]. In contrast Kjaergaard et al. found no relation between serum level of vitamin D and migraine although its lower level was related to higher nonmigraine type headache [15]. In a case report study by Prakash and Shah on 8 patients with chronic tension-type headache and vitamin D deficiency improvement in the headache was seen by administrating vitamin D [16]. Another study declared that headache is more common at higher latitudes which might be related to lower level of vitamin D in these areas [17]. Knutsen et al. in their study on 572 subjects in a multiethnic general practice in Norway concluded a high prevalence of hypovitaminosis D in cases with headache [18].

Several lines of evidence showed the role of an inflammatory process in pathophysiology of migraine. For instance, some studies demonstrated an increase of proinflammatory cytokines such as IL-6, IL-2, CGRP, and TNF in migraine [19]. Also some studies showed a relationship between migraine and inflammatory cytokine polymorphisms such as TNF-*α*-308G/A [20]. Gu et al. included five studies on Asian populations in their meta-analysis, including 985 cases and 958 controls. They revealed that the TNF-alpha −308G/A polymorphism was associated with migraine risk [21]. Yilmaz et al. in their survey suggested that the IL-1beta gene polymorphism contributes to migraine headache generation in migraine without aura patients [20].

A role for vitamin D is suggested in many of physiologic and pathologic mechanisms. 25-Hydroxyvitamin D deficiency is associated with inflammation through its effects on the secretion of cytokines such as interleukin-1 (IL-1), IL-2, IL-6, IL-12, TNF [5], and NO production [22].

The contribution of mast cells in migraine pathophysiology is implied [3], and we know the role of vitamin D in mast cell development and hormone content [23, 24]. These data support the hypothesis of an anti-inflammatory action of vitamin D in migraine as an inflammatory disease.

There are some studies indicating the role of endothelial dysfunction in the pathophysiology of migraine. A relation between cardiovascular diseases which are associated with endothelial dysfunction and migraine has been found [25].

Recent evidence has also linked migraine to a broader range of ischemic vascular disorders including cerebral ischemia stroke, angina, myocardial infarction, coronary revascularization, claudication, and cardiovascular mortality [26].

Since vitamin D has improved several diseases accompanying endothelial dysfunction (ED) and migraine is also associated with ED, another hypothesis is that vitamin D plays a role in the improvement of ED in migraine [27–29].

Recently, the relationship between polymorphisms of VDR, especially FokI and TaqI, and susceptibility to several diseases associated with inflammation such as diabetes, autoimmune diseases, and tuberculosis has been shown [5].

Mohammadnejad et al. demonstrated that allele t is an allele of risk for developing type 1 diabetes mellitus (TIDM) in the Iranian population [5]; also a meta-analysis showed that the FokI polymorphism may increase the risk of type 2 diabetes mellitus (T2DM) in East Asians [30].

Karray et al. found that FokI polymorphisms were significantly associated with rheumatoid arthritis (RA) and Behçet's disease [31]. Patients with Crohn's disease show more homozygotes for the TaqI polymorphism [32] against these data; in the other study only the f allele was higher in Crohn's patients compared with control [33].

A meta-analysis showed that the ff genotype has significant marginal association with tuberculosis although TaqI polymorphisms did not have such relation [34].

In nervous system diseases the role of VDR polymorphism is interesting because VDR is widely expressed in the human brain. Vitamin D may regulate inflammation in the brain. Lehmann et al. found that TaqI polymorphism accompanied Alzheimer's disease [35]. However, a study trying to associate Parkinson's disease to a TaqI polymorphism was unable to detect such a relation [36]. Also Huang and Xie, in a meta-analysis, found no relationship of multiple sclerosis (MS) with FokI and TaqI [37].

There is one study about migraine and VDR polymorphisms; Schürks et al. evaluated the association between 77 polymorphisms and migraine and demonstrated that VDR polymorphisms FokI and BsmI are not associated with migraine while TNF, CCR2, TGFB, NOS3, and IL9 polymorphisms can affect the susceptibility of having migraine [38]. This stands in contrast to our result that showed an association between FokI and migraine.

In conclusion our study showed the association of TaqI and FokI polymorphisms with migraine without aura; also headache severity in FokI heterozygote patients was more significant than in the homozygote patients. Our results were obtained from a small sample size and included only migraine patients without aura; therefore this is a preliminary conclusion and provides little evidence for a causal association between migraine and VDR polymorphisms. As in some other case-control studies we could not obtain enough information about an individual's exposure status over time, an important drawback. Another limitation concerns the selection of controls: we could not choose all of our controls to be fully matched with cases. Validation by a larger studies, especially randomized controlled trials, and cohort studies from a more diverse ethnic population is needed to confirm our findings.

## Figures and Tables

**Table 1:** The primer sequences of VDR gene polymorphisms.

SNP	Primer name	Sequences (5′-3′)
rs2228570	FokI forward	F: GTGGGTGGCACCAAGGAT
	FokI reverse	R: TCACAGGTCATAGCATTGAAGTG

rs731236	TaqI forward	F: GTGGGATTGAGCAGTGAG
	TaqI reverse	R: TTCTGGATCATCTTGGCATAG

VDR: vitamin D receptor, SNP: single nucleotide polymorphism.

**Table 2:** Demographic and clinical characteristics of patients with migraine.

Characteristic	Mean ± S.E. or percentage
Frequency of headache per month	8.32 ± 0.81
Family history for migraine	
Positive	77%
Negative	23%
Menstrual effect on the headache aggravation	
Yes	52%
No	48%
Total HIT-6 score	53.51 ± 1.93

HIT-6: headache impact test-6.

**Table 3:** Distribution of allele and genotype of FokIrs2228570 and TaqI rs731236 gene polymorphisms in the case and control groups.

	Case	Control	*P* value	OR (95% confidence interval)	RR (95% confidence interval)
Alleles					
FokI					
F	171 (83.1%)	185 (92.5%)	0.002	2.52 (1.33–4.78)	2.26 (1.27–4.01)
f	35 (16.9%)	15 (7.5%)			
TaqI					
T	154 (74.7%)	164 (82%)	0.038	1.53 (0.95–2.48)	1.40 (0.96–2.04)
t	52 (25.3%)	36 (18%)			
Genotypes					
Homozygote (FF)	68 (66.1%)	85 (85%)	0.001	2.91 (1.47–5.77)	2.26 (1.32–3.88)
Heterozygote (Ff)	35 (33.9%)	15 (15%)			
Homozygote (TT)	51 (49.6%)	64 (64%)	0.018	1.81 (1.03–3.18)	1.40 (1.01–1.93)
Heterozygote (Tt)	52 (50.4%)	36 (36%)			

OR: odds ratio, RR: relative risk.

**Table 4:** Distribution of vitamin D receptor haplotypes in the case and control groups.

	Case	Control	*P* value	OR (95% CI)	RR (95% CI)
Haplotypes					
TF	119 (66.8%)	149 (78.4%)	0.008	0.55 (0.34–0.88)	0.85 (0.75–0.96)
Tf	21 (11.8%)	10 (5.2%)	0.019	2.40 (1.10–5.26)	2.24 (1.08–4.62)
tF	38 (21.4%)	31 (16.4%)	0.135	1.39 (0.82–2.35)	1.30 (0.85–2.00)
*P* value	0.012				

OR: odds ratio, RR: relative risk, CI: confidence interval.

**Table 5:** Comparison of total HIT-6 score and frequency of headache per month between homozygote and heterozygote patients for FokI and TaqI genes.

	FokI		*P* value	TaqI		*P* value
	Homozygote (FF)	Heterozygote (Ff)		Homozygote (TT)	Heterozygote (Tt)	
Frequency of headache per month	8.54 ± 1.05	7.93 ± 1.30	0.361	7.94 ± 1.12	8.63 ± 1.17	0.338
Total HIT-6 score	49.87 ± 2.69	60.32 ± 1.87	0.004	50.86 ± 3.14	56.00 ± 2.29	0.093

Data are given as mean ± S.E.
